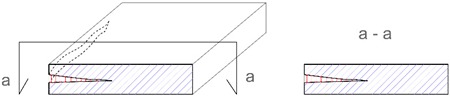# Non-Destructive Testing of Materials in Civil Engineering

**DOI:** 10.3390/ma12193237

**Published:** 2019-10-03

**Authors:** Krzysztof Schabowicz

**Affiliations:** Faculty of Civil Engineering, Wrocław University of Science and Technology, Wybrzeże Wyspiańskiego 27, 50-370 Wrocław, Poland; krzysztof.schabowicz@pwr.edu.pl

**Keywords:** non-destructive testing, diagnostic, acoustic methods, ultrasound, building materials, defects

## Abstract

This issue was proposed and organized as a means to present recent developments in the field of non-destructive testing of materials in civil engineering. For this reason, the articles highlighted in this editorial relate to different aspects of non-destructive testing of different materials in civil engineering, from building materials to building structures. The current trend in the development of non-destructive testing of materials in civil engineering is mainly concerned with the detection of flaws and defects in concrete elements and structures, and acoustic methods predominate in this field. As in medicine, the trend is towards designing test equipment that allows one to obtain a picture of the inside of the tested element and materials. Interesting results with significance for building practices were obtained.

## 1. Introduction

The current trend in the development of non-destructive testing of materials in civil engineering is mainly concerned with the detection of flaws and defects in concrete elements and structures, and acoustic methods predominate in this field. As mentioned in [1,2] much attention has been devoted to acoustic techniques because they have been greatly developed in recent years and there is a clear trend towards acquiring information about a tested element or structure from acoustic signals processed by proper software using complex data analysis algorithms. Another trend in the development of nondestructive techniques is the assessment of characteristics other than strength in elements or structures, particularly those made of concrete or reinforced concrete. As in medicine, the trend is towards designing test equipment that allows one to obtain a picture of the inside of the tested element. Increasingly, the offered apparatus is equipped with software based on sophisticated mathematical algorithms and artificial intelligence, which makes advanced analysis of the test results possible [2].

## 2. Non-Destructive Testing

**Figure 1:**
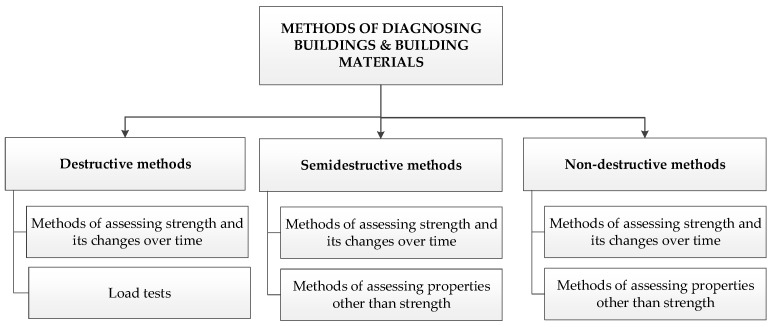
General classification of investigative methods useful for diagnosing buildings and building materials [2].

In construction, modern diagnostic methods are applied to building structural members and structures. Another major diagnostic field is the non-destructive testing of building materials. Such materials as wood, masonry units, concrete, fiber-cement and steel are subjected to tests for various reasons and at different times, e.g., during construction, but mainly during the service life. Many investigative methods are used for this purpose. Depending on the degree of their invasiveness, they can be divided into destructive, semi-destructive and non-destructive methods. A general classification of methods that are useful for diagnosing buildings and building materials [2] is presented in Figure 1.

Non-destructive methods are mainly used to test strength and investigate its changes over time. Usually samples taken from the structure, and sometimes whole members or structures, are tested in this way. Also load tests, which rather rarely are applied to buildings, but more often to bridges and roads, can be put into this category.

Semi-destructive and destructive methods are used to test samples and members. They can also be used to test whole structures. Strength and its changes over time are tested, but mainly other properties are tested in this way.

The difference between semi-destructive and non-destructive methods is that in the case of the former, the material is usually locally and superficially damaged when tested. No such damage occurs in the case of non-destructive methods. This is one of the reasons why they are suitable for testing large surfaces down to a considerable depth, and in general construction. Moreover, in the case of non-destructive methods, measurements can be repeated, whereby the test results can be verified and validated.

**Figure 2:**
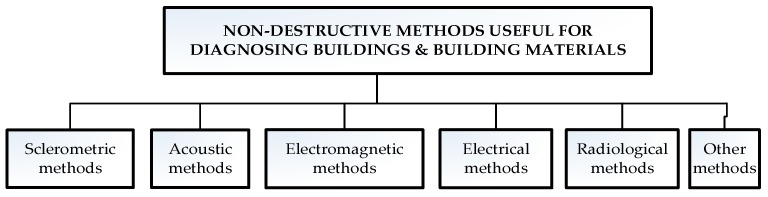
General classification of non-destructive methods useful for diagnosing buildings and building materials [3].

A general classification of non-destructive methods that are useful for diagnosing buildings and building materials [3] is presented in Figure 2. 

**Figure 3:**
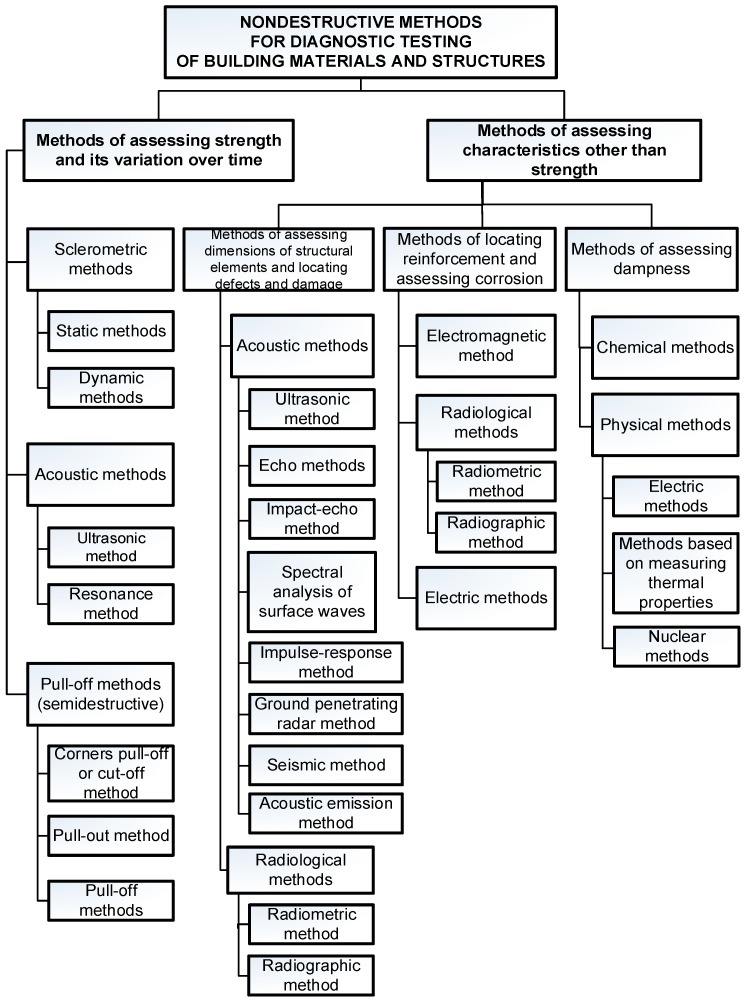
Non-destructive methods useful for diagnosing building structures and building materials [2,3].

Figure 3 shows a detailed classification of non-destructive methods useful for diagnosing buildings and building materials [2,3].

**Figure 4:**
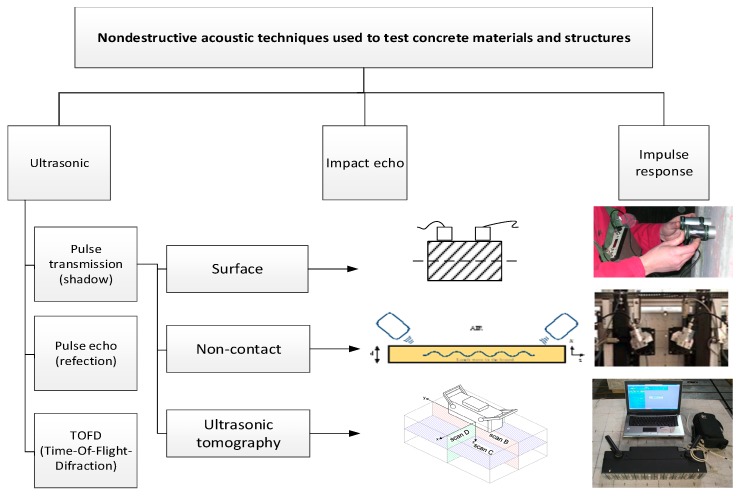
Classification of non-destructive acoustic methods suitable for testing concrete materials and structures [1,2].

**Figure 5:**
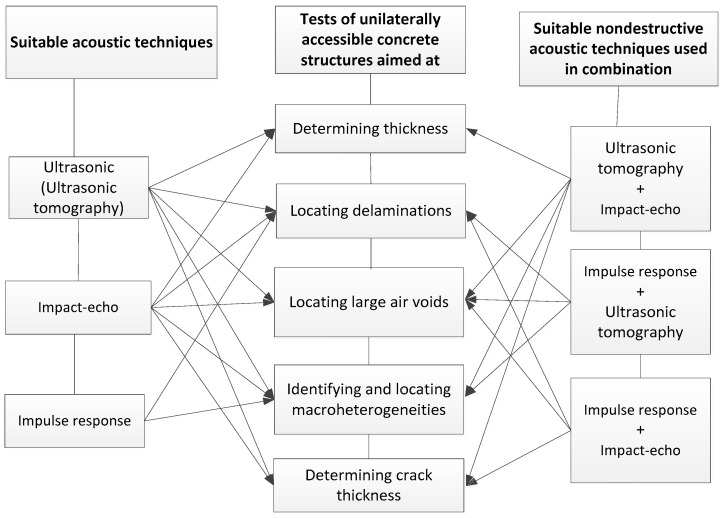
Suitability of state-of-the-art non-destructive acoustic methods, used individually or combined for testing concrete materials and structures [1].

Figure 4 shows a slightly modified classification (based on [2]) of the acoustic methods suitable for testing concrete materials and structures. This classification was presented in [1]. Figure 5 shows (on the basis of [1]) the suitability of the particular state-of-the-art non-destructive acoustic methods, used individually or combined for testing concrete and materials structures.

Table 1 lists, according to [1], the above (geometric and material) imperfections occurring in concrete structures, together with the terms proposed for their description and the assigned (as mentioned earlier) state-of-the-art non-destructive acoustic methods suitable for testing such structures [1].

The terms, i.e., identification, location, extent and intensity that are proposed in Table 1 for describing the imperfections are explained, using as an example the geometric imperfection in Table 2, and materials imperfection—delamination in Table 3. The proposed terms, based on tests carried out by the state-of-the-art non-destructive acoustic methods, make the description of the imperfections in concrete materials and structures more precise and, in my opinion, represent a scientific and research achievement [4,5,6,7,8,9,10,11,12,13,14].

## 3. Description of the Articles Presented in the Issue

In paper [15], a condition assessment of masonry pillars is presented. Non-destructive tests were performed on an intact pillar as well as three pillars with internal inclusions in the form of a hole, a steel bar grouted by gypsum mortar, and a steel bar grouted by cement mortar. The inspection utilized ultrasonic stress waves and the reconstruction of the velocity distribution was performed by means of computed tomography. The results showed the potential of tomographic imaging in characterizing the internal structure of pillars. Particular attention was paid to the assessment of the adhesive connection between a steel reinforcing bar embedded inside pillars, and the surrounding pillar body [15].

Paper [16] describes the validation of the following methods: semi-non-destructive, non-destructive, and ultrasonic technique for autoclaved aerated concrete (AAC). This study covers the compressive strength of AAC test elements with various density classes of 400, 500, 600, and 700 (kg/m^3^), at various moisture levels. Empirical data including the shape and size of specimens were established from tests on 494 cylindrical and cuboid specimens, and standard cube specimens using the general relationship for ordinary concrete (Neville’s curve). The developed methods turned out to be statistically significant and can be successfully applied during in-situ tests [16].

In paper [17], non-destructive tests of gantry cranes by means of the residual magnetic field (RMF) method were carried out for a duration of 7 years. The distributions of the residual magnetic field tangential and the normal components of their gradients were determined. A database of magnetograms was created. The results show that the gradients of tangential components can be used to identify and localize stress concentration zones in gantry crane beams. Special attention was given to the unsymmetrical distribution of the tangential component gradient on the surface of the crane beam. The anomaly was the effect of a slight torsional deflection of the beam as it was loaded. Numerical simulations with the finite element method (FEM) were used to explain this phenomenon. The displacement boundary conditions introduced into the simulations were established experimentally. Validation was carried out using the X-ray diffraction method, which confirmed the location of strain concentration zones (SCZs) identified by means of RMF testing [17].

Paper [18] presents the results of research aimed at identifying the degree of degradation of fiber-cement boards exposed to fire. The fiber-cement board samples were initially exposed to fire at various durations in the range of 1–15 min. The samples were then subjected to three-point bending and were investigated using the acoustic emission method. Artificial neural networks (ANNs) were employed to analyse the results yielded by the acoustic emission method. Fire was found to have a degrading effect on the fibers contained in the boards. As the length of exposure to fire increased, the fibers underwent gradual degradation, which was reflected in a decrease in the number of acoustic emission (AE) events recognized by the artificial neural networks as accompanying the breaking of the fibers during the three-point bending of the sample. It was shown that it is not sufficient to determine the degree of degradation of fiber-cement boards solely on the basis of bending strength (MOR) [18].

In paper [19] the aim of the experiment was to explore the possibility of using active thermography for testing large-sized building units (with high heat capacity) in order to locate material inclusions. As part of the experiment, two building partition models—one made of gypsum board (GB) and another made of oriented strand board (OSB)—were built. Three material inclusions (styrofoam, granite, and steel) with considerably different thermal parameters, were placed in each of the partitions. The distribution of the temperature field was studied on both sides of the partition for a few hours. The results showed that using the proposed investigative method, one can detect defects in building partitions under at least 22 mm of thick cladding. Active thermography can be used in construction for non-destructive materials testing. When the recording of thermograms is conducted for an appropriate length of time, inverse contrasts can be observed (on the same front surface) [19].

The aim of the study presented in [20] was to investigate the degradation of the microstructure and mechanical properties of fiber-cement board (FCB), which was exposed to environmental hazards, resulting in thermal impact on the microstructure of the board. The process of structural degradation was conducted under laboratory conditions by storing the FCB specimens in a dry, electric oven for 3 h at a temperature of 230 °C. Five sets of specimens, that differed in cement and fiber content, were tested. The fiber reinforcement morphology and the mechanical properties of the investigated compositions were identified both before, and after their carbonization. Visual light and scanning electron microscopy, X-ray micro tomography, flexural strength, and work of flexural test Wf measurements were used. The results obtained suggest a possible application of the UT method for an on-site assessment of the degradation processes occurring in fiber-cement boards [20].

Paper [21] presents procedures for investigating spun concrete and interpreting the results of such investigations, which make it possible to characterize the microstructure of the concrete. Three investigative methods were used to assess the distribution of the constituents in the cross section of the element: micro-computed tomography, 2D imaging (using an optical scanner) and nanoindentation. A procedure for interpreting and analysing the results is proposed. The procedure enables one to quantitatively characterize the following features of the microstructure of spun concrete: the mechanical parameters of the mortar, the aggregate content, the pore content, the cement paste content, the aggregate grading and the size (dimensions) of the pores. The proposed procedures constitute a valuable tool for evaluating the process of manufacturing spun concrete elements [21].

An enhanced singular value truncation method is proposed in paper [22] to evaluate structural damage more effectively by using a few lower order natural frequencies. The main innovations of the enhanced singular value truncation method lie in two aspects. The first is the normalization of linear systems of equations; the second is the multiple computations based on feedback evaluation. The proposed method is very concise in theory and simple to implement. Two numerical examples and an experimental example are employed to verify the proposed method. In the numerical examples, it was found that the proposed method can successively obtain more accurate damage evaluation results compared with the traditional singular value truncation method. In the experimental example, it was shown that the proposed method possesses more precise and fewer calculations compared with the existing optimization algorithms [22].

Article [23] presents results from non-destructive testing (NDT) that referred to the location and diameter or rebars in beam and slab members. The aim of this paper was to demonstrate that the accuracy and deviations of the NDT methods could be higher than the allowable execution or standard deviations. Tests were conducted on autoclaved aerated concrete beam and nine specimens that were specially prepared from lightweight concrete. The testing equipment was used to analyse how the rebar (cover) location affected the detection of their diameters and how their mutual spacing influenced the detected quantity of rebars. The considerations included the impact of rebar depth on cover measurements and the spread of obtained results. Tests indicated that the measurement error was clearly greater when the rebars were located at very low or high depths. Electromagnetic and radar devices were unreliable when detecting the reinforcement of small (8 and 10 mm) diameters at close spacing (up to 20 mm) and of large (20 mm) diameters at a close spacing and greater depths. Recommendations for practical applications were developed to facilitate the evaluation of a structure [23].

As mentioned in [24], predictable compressive strength of concrete is essential for concrete structure utilization and is the main feature of its safety and durability. Recently, machine learning has gained significant attention and future predictions for this technology are even more promising. Data mining on large sets of data has attracted attention because machine learning algorithms have achieved a level whereby they can recognize patterns that are difficult to recognize using human cognitive skills. In this paper, state-of-the-art achievements in machine learning techniques were utilized for concrete mix design. The authors prepared an extensive database of concrete recipes along with the relevant destructive laboratory tests, which was used to feed the selected optimal architecture of an artificial neural network. They translated the architecture of the artificial neural network into a mathematical equation that can be used in practical applications [24].

Paper [25] presents an assessment of the condition of wood from a wharf timber sheet wall after 70 years of service in a (sea)water environment. Samples taken from the structure’s different zones, i.e., the zone impacted by waves and characterized by variable water-air conditions, the zone immersed in water and the zone embedded in the ground were subjected to non-destructive or semi-destructive tests. Moreover, ultrasonic, stress wave and drilling resistance methods were used. Then, an X-ray microtomographic analysis was carried out. The results provided information about the structure of the material on the micro and macroscale and the condition of the material was assessed on that basis. Also, correlations between the particular parameters were determined. Moreover, the methods themselves were evaluated with regard to their usefulness for the in situ testing of timber and to estimate the mechanical parameters needed for the static load analysis of the whole structure [25].

In paper [26], the adsorption properties of waste brick dust (WBD) were studied by removal of PbII and CsI from an aqueous system. For adsorption experiments, 0.1 M and 0.5 M aqueous solutions of Cs^+^ and Pb^2+^ and two WBD (Libochovice (LB) and Tyn nad Vltavou (TN)) in the fraction below 125 m were used. The structural and surface properties of WBD were characterized by X-ray diffraction (XRD) in combination with solid-state nuclear magnetic resonance (NMR), supplemented by scanning electron microscopy (SEM), specific surface area (SBET), total pore volume and zero point of charge (pHZPC). LB was a more amorphous material showing better adsorption conditions than that of TN. The adsorption process indicated better results for Pb^2+^ due to the inner-sphere surface complexation in all Pb^2+^ systems, supported by the formation of insoluble Pb(OH)_2_ precipitation on the sorbent surface. A weak adsorption of Cs^+^ on WBD corresponded to the non-Langmuir adsorption run followed by the outer-sphere surface complexation. The leachability of Pb^2+^ from saturated WBDs varied from 0.001% to 0.3%, while in the case of Cs^+^, 4–12% of the initial amount was leached. Both LB and TN met the standards for PbII adsorption, yet completely failed for any CsI removal from water systems [26].

The aim of the study presented in [27] was to experimentally and numerically research the effects of the wave frequency on damage identification in a single-lap adhesive joint of steel plates. The ultrasonic waves were excited at one point of an analyzed specimen and then measured in a certain area of the joint. The recorded wave velocity signals were processed by way of a root mean square (RMS) calculation, giving the actual position and geometry of defects. In addition to the visual assessment of damage maps, a statistical analysis was conducted. The influence of an excitation frequency value on the obtained visualizations was considered in the wide range for a single defect. Supplementary finite element method (FEM) calculations were performed for three additional damage variants. The results revealed some limitations of the proposed method. The main conclusion was that the effectiveness of measurements strongly depends on the chosen wave frequency value [27].

As mentioned in [28], the nondestructive testing of reinforced concrete chimneys, especially the high ones is an important element in the assessment of their condition, making it possible to forecast their safe service lifespan. Industrial chimneys are often exposed to the strong action of acidic substances—they are adversely affected by the flue gas condensate on the inside and by acid precipitation on the outside. During the service life of such chimneys, their condition should be monitored in order to prevent structural failures and indicate the most endangered parts of the structure. The methods for the interpretation of results from thermovision studies to determine the safety and durability of industrial chimneys are shown in [28].

In paper [29], the authors present two methods used in the identification of viscoelastic parameters of asphalt mixtures used in pavements. The static creep test and the dynamic test, with a frequency of 10 Hz, were carried out based on the four-point bending beam (4BP). In the method for identifying viscoelastic parameters for the Brugers’ model, they included the course of a creeping curve (for static creep) and fatigue hysteresis (for dynamic test). It was shown that these parameters depend significantly on the load time, method used, and temperature and asphalt content. A similar variation in the parameters depending on temperature was found for the two tests, but different absolute values were obtained. The authors also found that the parameters should be determined using the creep curve for the static analyses with persistent load, whereas in the case of the dynamic studies, the hysteresis is more appropriate. In the 4BP dynamic test, the authors determined the relationships between damping and viscosity coefficients, showing that material variability depends on the test temperature [29].

Article [30] presents the application of the acoustic emission technique (AE) in order to detect the initiation and examination of the crack growth process in steel used in engineering construction. The tests were carried out on specimens with a single edge notch in bending (SENB) made of 40CrMo steel. Crack opening displacement, force parameter and potential drop signal were measured in the tests. During loading of the specimens, the fracture mechanism was classified as brittle. Accurate investigations of the cracking process by the acoustic emission (AE) method and observation of fracture surfaces by scanning electron microscopy (SEM) have made it possible to determine that the cracking process is more complex than the classically understood brittle fracture. The work focuses on the comparison of selected parameters of the acoustic emission signals in the phase before the initiation and development of brittle fracture cracking [30].

Paper [31] examines the repair of a stand of a motorbike speedway stadium. The synchronized dancing of fans cheering during a meeting brought the stand into excessive resonance. The main goal of this research was to propose a method for the structural tuning of stadium stands. Non-destructive testing by vibration methods was conducted on a selected stand segment, the structure of which recurred in the remaining stadium segments. Through experiments, the authors determined the vibration forms throughout the stand, taking into account the dynamic impact of fans. Numerical analyses were performed on the 3-D finite element method (FEM) stadium model to identify the dynamic jump load function. The results obtained on the basis of sensitivity tests using the finite element method allowed the tuning of the stadium structure to successfully meet the requirements of the serviceability limit state [31].

In paper [32] the author asked a question: is the variation in the compressive strength of concrete across the thickness of horizontally cast elements negligibly small, or rather, does it need to be taken into account at the design stage? In order to determine if the compressive strength of concrete varies across the thickness of horizontally cast elements, ultrasonic tests and destructive tests were carried out on core samples taken from a 350 mm thick slab made of class C25/30 concrete. Special point-contact probes were used to measure the time taken for the longitudinal ultrasonic wave to pass through the tested sample. The correlation between the velocity of the longitudinal ultrasonic wave and the compressive strength of the concrete in the slab was determined It was found that the destructively determined compressive strength varied only slightly (by 3%) across the thickness of the placed layer of concrete, whereas the averaged ultrasonically determined strength of the concrete in the same samples does not vary across the thickness of the analyzed slab. Therefore, it was concluded that the slight increase in concrete compressive strength with depth below the top surface is a natural thing and need not be taken into account in the assessment of the strength of concrete in the structure [32].

Paper [33] presents the results of investigations into the effect of freeze-thaw cycling on the failure of fiber-cement boards and on the changes taking place in their structure. An artificial neural network was employed to analyze the results yielded by the acoustic emission method. The investigations conclusively proved that freeze-thaw cycling has an effect on the failure of fiber-cement boards, as indicated mainly by the fall in the number of AE events recognized as accompanying the breaking of fibers during the three-point bending of the specimens. SEM examinations were carried out to get a better insight into the changes taking place in the structure of the tested boards. Interesting results with significance for building practice were obtained [33].

The findings of the study in article [34] deepen the understandings of pore structure damage in cement-based materials (CBMs) by mercury intrusion, and provide methodological insights into the microstructure characterization of CBMs by XCT. Mercury intrusion porosimetry (MIP) is questioned for possibly damaging the micro structure of CBMs, but this theme still has a lack of quantitative evidence. By using X-ray computed tomography (XCT), this study reported an experimental investigation of the pore structure damage in paste and mortar samples after a standard MIP test. XCT scans were performed on the samples before and after mercury intrusion. Because of its very high mass attenuation coefficient, mercury can greatly enhance the contrast of XCT images, providing a path to probe the same pores with and without mercury fillings. The paste and mortar showed different MIP pore size distributions but similar intrusion processes. A grey value inverse for the pores and material skeletons before and after MIP was found. With the features of excellent data reliability and robustness verified by a threshold analysis, the XCT results characterized the surface structure of voids, and diagnosed the pore structure damages in terms of pore volume and size of the paste and mortar samples [34]. 

Paper [35] presents the identification of the destruction process in a quasi-brittle composite based on acoustic emission and the sound spectrum. The tests were conducted on a quasi-brittle composite. The sample was made from ordinary concrete with dispersed polypropylene fibers. The possibility of identifying the destruction process based on the acoustic emission and sound spectrum was confirmed and the ability to identify the destruction process was demonstrated. Three- and two-dimensional spectra were used to identify the destruction process. The three-dimensional spectrum provides additional information, enabling a better recognition of changes in the structure of the samples on the basis of the analysis of sound intensity, amplitudes, and frequencies. The paper shows the possibility of constructing quasi-brittle composites to limit the risk of catastrophic destruction processes and the possibility of identifying those processes with the use of acoustic emission at different stages of destruction [35].

The aim of the study in article [36] is to investigate two moisture monitoring techniques to promote moisture safety in wood-based buildings (i.e., new structures, as well as renovated and protected buildings). The study is focused on the comparison of two electrical methods that can be employed for the non-destructive moisture monitoring of wood components integrated in the structure of buildings. The main principle of the two presented methods of measuring the moisture by electric resistance is based on a simple resistor–capacitor (RC) circuit system improved with an ICM7555 chip and integrator circuit using a TLC71 amplifier. The RC-circuit is easier to implement thanks to the digital signals of the used chip, whilst the newly presented integration method allows faster measurement at lower moisture contents. A comparative experimental campaign utilizing spruce wood samples was conducted in this regard. Based on the results obtained, both methods can be successfully applied to wood components in buildings for moisture contents above 8% [36]. 

The methodology of multi-scale structural assessment of the different cellulose fiber-cement boards subjected to high temperature treatment was proposed in article [37]. Two specimens were investigated: Board A (air-dry reference specimen) and Board B (exposed to a temperature of 230 °C for 3 h). At macroscale all considered samples were subjected to the three-point bending test. Next, two methodologically different microscopic techniques were used to identify evolution (caused by temperature treatment) of geometrical and mechanical morphology of boards. For that purpose, SEM imaging with EDS analysis and nanoindentation tests were utilized. High temperature was found to have a degrading effect on the fibers contained in the boards. Most of the fibers in the board were burnt-out, or melted into the matrix, leaving cavities and grooves which were visible in all of the tested boards. Nanoindentation tests revealed significant changes of mechanical properties caused by high temperature treatment: “global” decrease of the stiffness (characterized by nanoindentation modulus) and “local” decrease of hardness. The results observed at microscale are in a very good agreement with macroscale behavior of considered composite. It was shown that it is not sufficient to determine the degree of degradation of fiber-cement boards solely on the basis of bending strength; advanced, microscale laboratory techniques can reveal intrinsic structural changes [37]. 

In paper [38], the ultrasonic method using exponential heads with spot surface of contact with the material was chosen for the measurements of concrete strength in close cross sections parallel to the corroded surface. The test was performed on samples taken from compartments of a reinforced concrete tank after five years of operation in a corrosive environment. Test measurements showed heterogeneity of strength across the entire thickness of the tested elements. It was determined that the strength of the elements in internal cross sections of the structure was up to 80% higher than the initial strength. A drop in the mechanical properties of concrete was observed only in the close zone near the exposed surface. The dependence of the compressive strength of standard cubic samples on the duration of their exposure in the sulphate corrosion environment has been described [38].

The ultrasonic pulse velocity test, the rebound hammer test is the most common NDT method currently used for this purpose. However, estimating compressive strength using general regression models can often yield inaccurate results. The experiment results in paper [39] show that the compressive strength of any concrete can be estimated using one’s own newly created regression model. A traditionally constructed regression model can predict the strength value with 50% reliability, or when two-sided confidence bands are used, with 95% reliability. However, civil engineers usually work with the so-called characteristic value defined as a 5% quantile. Therefore, it seems appropriate to adjust conventional methods in order to achieve a regression model with 95% one-sided reliability. This paper describes a simple construction of such a characteristic curve. The results show that the characteristic curve created for the concrete in question could be a useful tool, even outside of practical application [39].

## 4. Conclusions

As mentioned at the beginning, this issue was proposed and organized as a means of presenting recent developments in the field of non-destructive testing of materials in civil engineering. For this reason, the articles highlighted in this editorial relate to different aspects of non-destructive testing of materials in civil engineering, from building materials to building structures. Interesting results with significance for the materials were obtained and all of the papers have been precisely described.

## Figures and Tables

**Table 1:** Selected imperfections occurring in concrete structures, together with terms proposed for their description and assigned state-of-the-art non-destructive acoustic methods suitable for testing such structures [1].

Type/Description of Imperfection		Test Method					
		Ultrasonic Tomography Method	Impact-Echo Method	Impulse Response Method	Ultrasonic Tomography + Impact Echo	Impulse Response + Ultrasonic Tomography	Impulse Response + Impact-Echo
Incorrect thickness of member	Identification	●	●	-	●	-	-
	Location	●	●	-	●	-	-
	Extent	●	●	-	●	-	-
	Intensity	○	○	-	○	-	-
Delamination	identification	●	●	●	●	●	●
	Location	●	●	○	●	●	●
	Extent	●	●	●	●	●	●
	Intensity	-	○	-	○	-	○
Large air voids	Identification	●	●	-	●	●	●
	Location	●	●	-	●	●	●
	Extent	●	●	-	●	●	●
	Intensity	N.A.	N.A.	N.A.	N.A.	N.A.	N.A.
Zones of concrete macroheterogeneities	Identification	●	●	●	●	●	●
	Location	●	●	●	●	●	●
	Extent	●	●	○	●	●	●
	Intensity	-	○	-	○	-	○
Cracks	Identification	●	●	-	●	-	●
	Location	●	-	-	●	-	-
	Extent	-	●	-	●	-	●
	Intensity	-	●	-	●	-	●

Symbols: **●**—suitable method, **○**—partially suitable method, **-**—unsuitable method, N.A.—not applicable.

**Table 2:** Explanation of proposed terms for describing geometric imperfection in concrete structures tested by non-destructive acoustic methods.

Description of Imperfection	Illustration of Imperfection Description	
	View	Cross Section
Identification An imperfection (incorrect thickness of the structure) is found to be present.	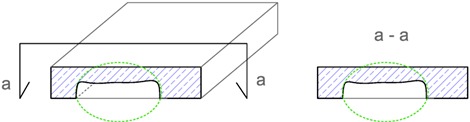	
Location The place of occurrence of the imperfection in the cross section of the structure is determined with accuracy dependent on the equipment used.	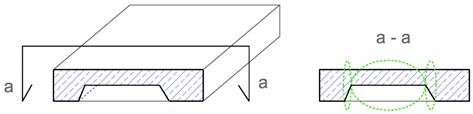	
Extent The size (surface area and volume) of the place where the imperfection occurs is determined with accuracy dependent on the equipment used.	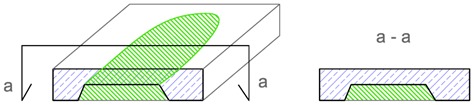	
Intensity The degree of advancement of the imperfection (the distribution of locally incorrect thickness in the whole place of its occurrence).	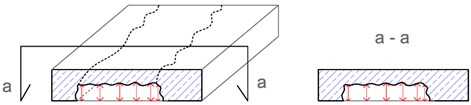	

**Table 3:** Explanation of proposed terms for describing materials imperfection—delamination in concrete structures tested by non-destructive acoustic methods.

Description of Imperfection	Illustration of Imperfection Description	
	View	Cross Section
Identification An imperfection (incorrect thickness of the structure) is found to be present.	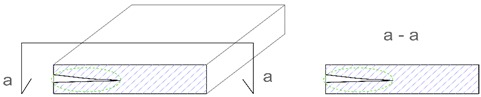	
Location The place of occurrence of the imperfection in the cross section of the structure is determined with accuracy dependent on the equipment used.	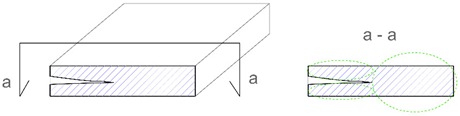	
Extent The size (surface area and volume) of the place where the imperfection occurs is determined with accuracy dependent on the equipment used.	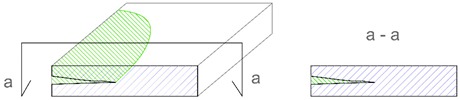	
Intensity The degree of advancement of the imperfection (the distribution of locally incorrect thickness in the whole place of its occurrence).